# Epidermal growth factor receptor inhibition with Gefitinib does not alter lung responses to mechanical ventilation in fetal, preterm lambs

**DOI:** 10.1371/journal.pone.0200713

**Published:** 2018-07-13

**Authors:** T. Brett Kothe, Emily Royse, Matthew W. Kemp, Haruo Usuda, Masatoshi Saito, Gabrielle C. Musk, Alan H. Jobe, Noah H. Hillman

**Affiliations:** 1 Division of Neonatology, Cardinal Glennon Children’s Hospital, Saint Louis University, Saint Louis, Missouri, United States of America; 2 School of Women’s and Infants’ Health, University of Western Australia, Perth, Western Australia, Australia; 3 Centre for Perinatal and Neonatal Medicine, Tohoku University Hospital, Sendai, Japan; 4 Animal Care Services, University of Western Australia, Perth, Western Australia, Australia; 5 Division of Pulmonary Biology, Cincinnati Children’s Hospital Medical Center, University of Cincinnati, Cincinnati, Ohio, United States of America; University of Giessen Lung Center, GERMANY

## Abstract

**Background:**

Epidermal growth factor receptor (EGFR) is important for airway branching and lung maturation. Mechanical ventilation of preterm lambs causes increases in EGFR and EGFR ligand mRNA in the lung. Abnormal EGFR signaling may contribute to the development of bronchopulmonary dysplasia.

**Hypothesis:**

Inhibition of EGFR signaling will decrease airway epithelial cell proliferation and lung inflammation caused by mechanical ventilation in preterm, fetal sheep.

**Methods:**

Following exposure of the fetal head and chest at 123±1 day gestational age and with placental circulation intact, fetal lambs (n = 4-6/group) were randomized to either: 1) Gefitinib 15 mg IV and 1 mg intra-tracheal or 2) saline IV and IT. Lambs were further assigned to 15 minutes of either: a) Injurious mechanical ventilation (MV) or b) Continuous positive airway pressure (CPAP) 5 cmH_2_O. After the 15 minute intervention, the animals were returned to the uterus and delivered after i) 6 or ii) 24 hours in utero.

**Results:**

MV caused lung injury and inflammation, increased lung mRNA for cytokines and EGFR ligands, caused airway epithelial cell proliferation, and decreased airway epithelial phosphorylated ERK1/2. Responses to MV were unchanged by Gefitinib. Gefitinib altered expression of EGFR mRNA in the lung and liver of both CPAP and MV animals. Gefitinib decreased the liver SAA3 mRNA response to MV at 6 hours. There were no differences in markers of lung injury or inflammation between CPAP animals receiving Gefitinib or saline.

**Conclusion:**

Inhibition of the EGFR pathway did not alter acute lung inflammation or injury from mechanical ventilation in preterm sheep.

## Introduction

Bronchopulmonary dysplasia (BPD), which affects up to 40% of very low birth weight preterm infants, is characterized by alveolar simplification, pulmonary microvascular and airway epithelial injury [1–4]. School-age children with a history of moderate to severe BPD have decreased FEV_1_, increased respiratory symptoms, and decreased peak flow measurements [1, 3, 5, 6]. Lung inflammation resulting from mechanical ventilation is central to the development of the airway alterations and the distal lung simplification of BPD [4, 7, 8]. Sheep and human lungs have similar airway epithelial cell types and distributions in the peripheral lung, thus sheep provide a useful model for evaluating lung and airway injury [9–11]. Mechanical ventilation in preterm sheep stretches the airways, causes airway epithelial injury and proliferation, increases α-smooth muscle actin around airways, and causes diffuse lung inflammation and maturation [12–15]. Preterm fetal sheep repair the epithelial injury through activation of basal cells in the bronchioles and club cells in the terminal bronchiole, but excessive proliferation may contribute to the small airway disease in BPD [3, 15]. Since the majority of infants born at 28 weeks gestation or less will receive mechanical ventilation, it is important to identify therapies to decrease the lung inflammation and airway alterations [16].

Epidermal growth factor receptor (EGFR) activation is critical for lung development and the pathology of multiple lung diseases [17–20]. Mice with inactivated EGFR are born with hypoplastic lungs that have impaired branching morphogenesis, deficient alveolarization and septation, and type II pneumocyte immaturity [20]. In addition to its role in development, EGFR ligands mediate smooth muscle changes and airway hyper-reactivity [21, 22], cause basal cell proliferation in human epithelial cultures [23], and EGFR is necessary for basal cell proliferation in mice [24]. EGFR pathways also regulate the proliferation and trans-differentiation of club cells during re-epithelialization of injured airways in transgenic mice [9, 24, 25]. Though EGFR activation is required for normal mucin production, over-activation can lead to mucus cell hyperplasia through cellular differentiation into goblet cells [26, 27], which may also contribute to the BPD phenotype. Inhibition of EGFR signaling can decrease the inflammation and airway responses in mouse models of asthma [18, 28]. Acute lung injury from LPS exposure and mechanical ventilation is also decreased with EGFR inhibition [17, 19].

Prior studies have demonstrated that mechanical ventilation of preterm sheep increased mRNA for EGFR and the EGFR ligands amphiregulin (AREG), epiregulin (EREG), and heparin-binding EGF (HB-EGF) in the peripheral lung [15, 29]. Intra-amniotic exposure to E. coli LPS or Ureaplasma did not change the increases of ventilation-induced EGFR and ligand mRNA [29]. We used a fetal sheep model, which maintains placental support during injurious ventilation and allows return of the fetus to the uterus, to evaluation of the progression of injury and repair for 6 or 24 hours [13, 30, 31]. Using the EGFR inhibitor Gefitinib, given both systemically and locally to the airways, we tested the hypothesis that EGFR signaling promotes the lung inflammation, bronchiolar cell proliferation, and increased acute-phase activation caused by mechanical ventilation of preterm, fetal sheep. Systemic responses to mechanical ventilation and EGFR inhibition were evaluated in the liver.

## Methods

All animal experiments were performed with the approval of the Animal Ethics Committee of the University of Western Australia.

### Maternal anesthesia and fetal exteriorization

Date-mated Merino Ewes at 123 ± 1 days gestational age (GA; term is about 150 days GA) were pre-medicated with a combination of acepromazine 0.03 mg/kg and buprenorphine 0.02 mg/kg IM 45 minutes prior to induction of anaesthesia with ketamine 5 mg/kg, and midazolam 0.25 mg/kg IV. The trachea was intubated and anaesthesia was maintained with isoflurane (1.5–2% in 100% O_2_), which crosses the placenta, anesthetizes the fetus, and eliminates fetal breathing [30]. Ropivacaine was injected at the surgery site along the ventral midline (1 mg/kg SC). After a midline hysterotomy, the fetal head and chest were exteriorized to the umbilicus to allow for lung expansion that was not inhibited by intra-uterine pressures. Fetal blood flow was maintained [13]. A tracheostomy was performed on the fetal lamb, and a 4 French cuffed endotracheal tube (ETT) was placed and secured. Fetal lung fluid (FLF) was then removed via the ETT using a syringe and suction catheter and an aliquot of the FLF was snap frozen.

### Fetal interventions

Fetal lambs were randomized to one of eight fetal intervention groups (n = 4-6/group). Lambs were first randomized to receive treatment (IV and intra-tracheal) with either 1) Gefitinib or 2) Saline. Gefitinib treated lambs received Gefitinib 15 mg in 2 mL saline:DMSO mixed 1:1 (5 mg/kg based on estimated fetal weight) IV via the jugular vein and b) Gefitinib 1 mg in 15 mL saline:DMSO (1:15, approximately 20μM concentration in fetal lung fluid, Cayman Chemical, USA) washed up and down via the ETT three times. Saline lambs received: a) saline (2 mL saline:DMSO mixed 1:1) IV and 15 mL saline:DMSO (mixed 1:15) washed up and down via the ETT three times. These interventions were performed immediately after exteriorization, prior to any ventilation or CPAP.

The fetal lambs were then assigned to receive either 15 minutes of: a) Continuous positive airway pressure (CPAP) at 5 cmH_2_O, or b) mechanical ventilation with escalating tidal volumes. Each animal was positioned prone on the abdomen of the ewe to maintain placental circulation. For lambs receiving injurious mechanical ventilation, a Fabian ventilator (Acutronic, Switzerland) was used with initial settings of a rate of 40 breaths/minute, inspiratory time of 0.7 seconds, positive end expiratory pressure of 0 cmH_2_O, and an initial peak inspiratory pressure of 40 cmH_2_O. The weight of the animal was estimated, and peak inspiratory pressure was adjusted (max 55 cmH_2_O) to achieve a target of 5 mL/kg tidal volume by 5 minutes of ventilation, 10 mL/kg by 10 minutes, and 15 mL/kg between 10–15 minutes [12, 13]. Ventilator variables were chosen to induce airway and peripheral lung injury [13], and the animals received 100% N_2_ to avoid oxygen exposure. After the 15-minute intervention, the animals had the ETT removed, the trachea was ligated to prevent loss of Gefitinib or saline from the lungs, and the fetal lambs were returned to the uterus, with delivery and tissue collection 6 or 24 hours later.

### Tissue sampling

Postmortem, inflation and deflation pressure-volume curves were measured with stepwise changes in pressure to a maximum of 40 cmH_2_O [32]. Bronchoalveolar lavage fluid (BALF) was collected by repetitive saline lavage of the left lung and was used for cell counts. Cytospins were stained with Diff Quick (Fischer Scientific, USA) for differential cell counts on 200 cells/slide [32]. Tissues from the trachea, right lower lobe (peripheral lung), left mainstem bronchus with surrounding lung parenchyma removed, and liver were snap frozen for RNA isolation [33]. The right upper lobe of lung was inflation fixed at 30 cmH_2_O with 10% formalin and then paraffin embedded [33].

### Quantitative RT-PCR

Messenger RNA (mRNA) was extracted from the left mainstem bronchus, the peripheral lung tissue of the right lower lobe, and the liver with TRIzol (Invitrogen, USA). cDNA was produced from 1 μg mRNA using the Verso cDNA kit (Thermoscientific, USA). Custom Taqman gene primers (Life Technologies) for ovine sequences for amphiregulin (AREG), epiregulin (EREG), epidermal growth factor receptor (EGFR), ErbB2, ErbB3, ErbB4, keratin 5 (KRT5), KRT8, KRT14, Interleukin 1ß (IL-1β), IL-8, monocyte chemoattractant protein-1 (MCP-1), mucin 5B (MUC5B), p63 and serum amyloid A3 (SAA3) [29, 34]. Quantitative RT-PCR was performed with iTaq Universal mix (Bio-Rad) in a 15 μL reaction on a CFX Connect machine and software (Bio-Rad). 18S primers (Life Technologies, USA) were used as the internal loading control.

### Immunohistochemistry

Immunohistochemistry protocols used paraffin sections (4 μm) of formalin-fixed tissues that were antigen retrieved in heated citrate and pretreated with 3% hydrogen peroxide to inactivate endogenous peroxidases [35, 36]. The sections were incubated with anti-human Ki67 at 1:100 dilution (Thermoscientific, USA) or anti-human phosphorylated ERK1/2 at 1:100 dilution (R&D Systems, USA) in 4% normal horse serum, followed by a biotin labeled secondary antibody. Immunostaining was visualized by Vectastain ABC Peroxidase Elite kit (Vector Laboratories, USA). The antigen detection was enhanced with nickel-DAB, followed by TRIS-cobalt, and the nuclei counterstained with nuclear fast red [36]. Blinded slides had airway epithelial cells counted (10 airways per animal) for positive staining with pERK1/2 or Ki67, and reported as percent positive cells. Hematoxylin and Eosin stained tissues were blinded and evaluated for airway injury and inflammation, as previously described [37].

### Western blot analysis

Protein from lung homogenates (30 μg protein, denatured, phosphorylase treated) were run on Tris-glycine 10% (Bio-Rad, USA) gel and transferred to 0.45μm nitrocellulose cells (Bio-Rad, USA). Membranes were incubated with rabbit anti-pERK1/2 (MAB10108) 1:250 (R&D systems, USA) or rabbit anti-β actin (PA1-21167) 1:250 (ThermoScientific, USA) overnight then IgG-HRP secondary antibodies were applied (1:10000). Membranes were developed with Immobilon HRP Substrate (Millipore, USA), imaged on PXi Imager (Syngene, USA), and quantified (ImageJ, USA). pERK/ β actin was analyzed, with results reported as fold increase over control.

### Data analysis and statistics

Results are shown as mean ± SEM, reported as fold increase over Saline CPAP 6 Hour animals to control for effects of the surgery and intubation procedures. Statistics were analyzed using Prism 6 (GraphPad, USA) by using Student’s *t*-test, Mann-Whitney non-parametric, or ANOVA tests as appropriate. Significant was accepted as p < 0.05.

## Results

The majority of the fetuses survived the intervention and recovery after return to the uterus, but two fetuses, one in the 24-hour saline and one in the 24-hour gefitinib, were dead at delivery. There were no differences in gestational age (123 ± 1 days), gender, or birth weight among the groups. All animals assigned to ventilation groups received similar tidal volumes at 5, 10, and 15 minutes (Table 1). CPAP groups received a PEEP of 5 cmH_2_O without any tidal volumes. All the ventilated animals required the maximum value of 55 cmH_2_O because the tidal volumes were lower than the target of 15 mL/kg (Table 1). The low lung compliance of the fetal lambs was due to prematurity and surfactant deficiency. The fetal chest was entirely exteriorized, and thus lung expansion was not inhibited by compression of the chest. The postmortem volumes at 40 cmH_2_O pressure (Table 1, V40) were lower in fetal lambs that received mechanical ventilation compared to CPAP animals, consistent with lung injury and inflammation. On histologic exam of the lungs, ventilation caused lung inflammation, edema, and epithelial sloughing as measured by the elevated histologic injury scores (Table 1, Injury scores) compared to lambs receiving CPAP. There were no differences between lambs receiving Gefitinib or saline in any of these measures.

**Table 1:** Characteristics and ventilation variables.

Group	N	BW	V_T_/kg (mL)			V40	Injury Score
		(kg)	5 min	10 min	15 min	(mL/kg)	Out of 8
**Saline CPAP 6h**	5	2.7	--	--	--	8.0±2.9	2.5±0.5
**Gefitinib CPAP 6h**	5	3.0	--	--	--	6.6±0.7	2.5±0.6
**Saline CPAP 24h**	4	3.1	--	--	--	15.9±9.1	2.9±0.3
**Gefitinib CPAP 24h**	5	3.2	--	--	--	14.1±3.9	2.2±0.4
**Saline Vent 6h**	6	2.8	5.7±0.2	9.3±0.6	11.1±0.5	4.5±0.4*	6.6±0.2*
**Gefitinib Vent 6h**	6	3.0	5.5±0.4	8.9±0.3	9.8±0.6	3.5±0.1*	6.4±0.2*
**Saline Vent 24h**	5	2.9	5.2±0.2	8.9±0.8	10.3±1.3	3.9±0.2*	6.6±0.5*
**Gefitinib Vent 24h**	6	3.0	5.4±0.3	8.8±0.7	9.7±0.9	4.4±0.3*	6.5±0.2*

* p < 0.05 vs Saline CPAP 6h

### EGFR ligands and acute phase mRNA

**Fig 1:**
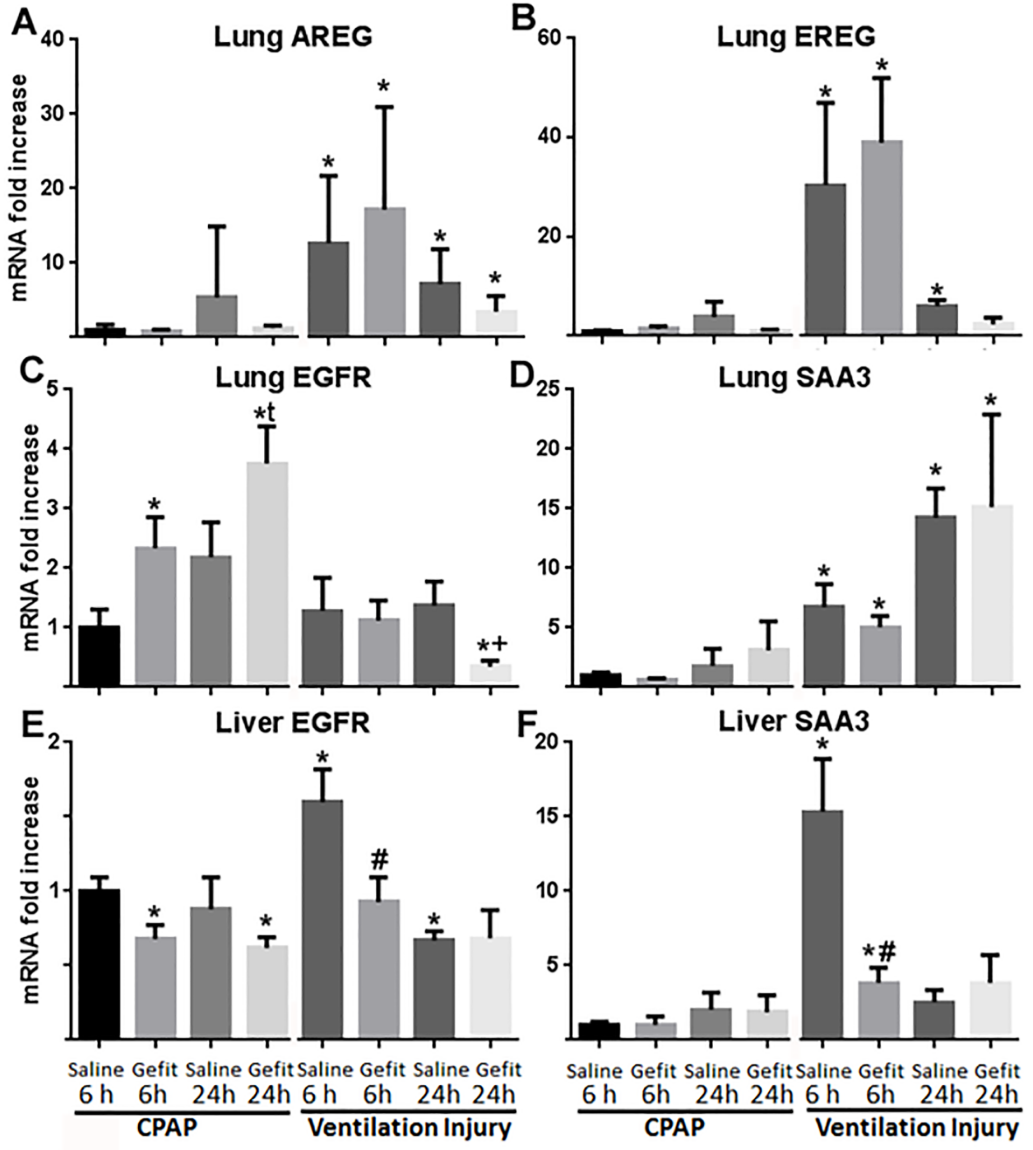
mRNA values for lung (A-D) and liver (E-F). (A) Amphiregulin (AREG), (B) Epiregulin (EREG), (C) Epidermal growth factor receptor (EGFR), and (D) Serum Amyloid A3 (SAA3) mRNA in the peripheral lung tissue. (E) EGFR mRNA and (F) SAA3 mRNA in the liver. mRNA values compared to SC6, whose mean is set to 1. Mean ± SEM. * = p <0.05 vs SC6; t = p<0.05 vs SC24; # = p<0.05 vs SV6; + = p< 0.05 vs SV24.

Mechanical ventilation, but not CPAP, increased the mRNA for EGFR ligands AREG (Fig 1A) and EREG (Fig 1B) in the peripheral lung tissue at 6 and 24 hours. These results were not affected by Gefitinib. Gefitinib increased EGFR mRNA in CPAP lambs at 6 and 24 hours compared to saline (Fig 1C). Gefitinib decreased EGFR mRNA at 24 hours in mechanically ventilated lambs (Fig 1C). The acute phase response serum amyloid A3 (SAA3) was increased in the lung with mechanical ventilation but these changes were not affected by Gefitinib (Fig 1D). In the liver, Gefitinib decreased EGFR mRNA in animals receiving CPAP at 6 and 24 hours (Fig 1E). Liver EGFR was increased by mechanical ventilation at 6 hours in animals receiving saline, but unchanged in animals receiving Gefitinib (Fig 1E). Liver SAA3 mRNA increased in animals receiving mechanical ventilation at 6 hours, and was decreased 4 fold by Gefitinib at same time point (Fig 1F).

### Airway epithelial cell proliferation and cell types

**Fig 2:**
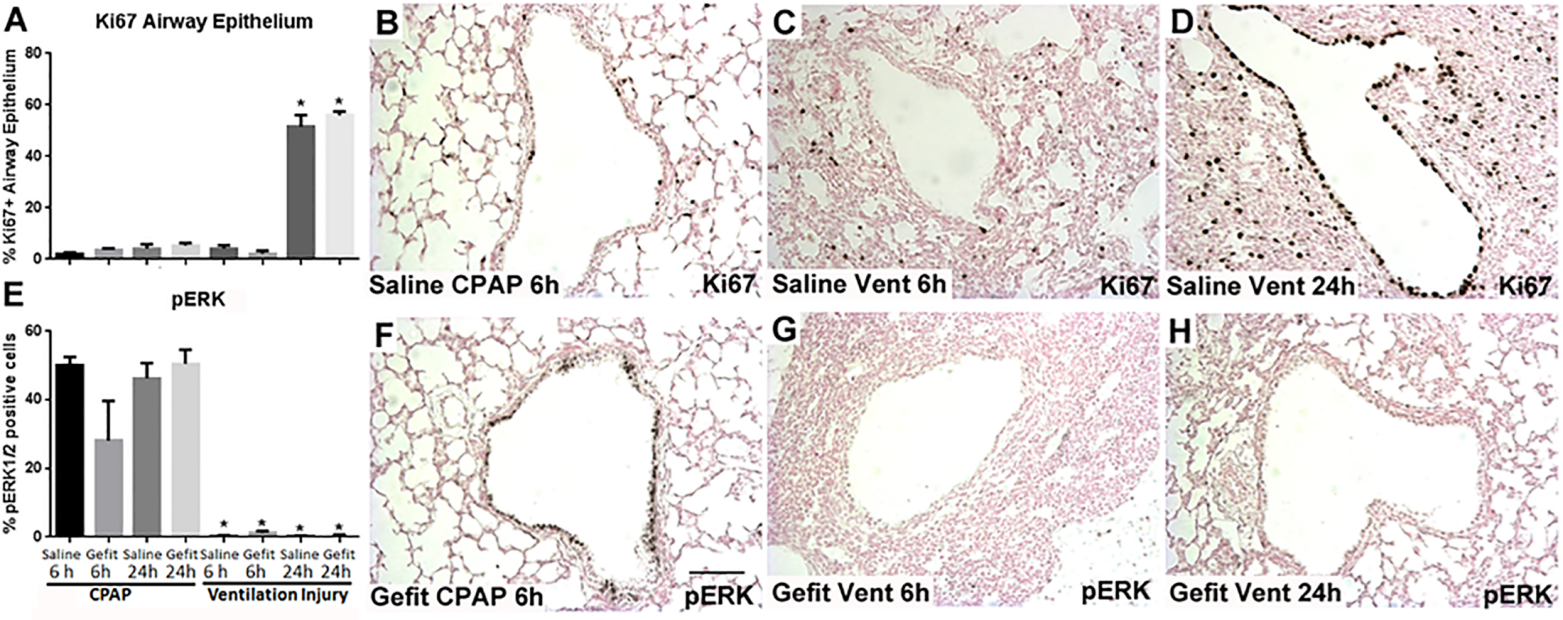
Airway epithelial proliferation (Ki67) and pERK1/2. (A-D Ki67): (A) Percent of airway epithelial cells that are Ki67 positive. (B) Saline CPAP 6 hour animals (SC6) have minimal staining. (C) Saline Ventilation 6 hour (SV6) animals have increased lung congestion without increased Ki67 signal in the airways. (D) SV24 animals are Ki67+ in the majority of epithelial cells. No effect of Gefitinib is observed. (E-H pERK 1/2): (E) phosphorylated ERK1/2 is present in the majority of airway epithelial cells in CPAP lambs, but absent from lambs receiving mechanical ventilation. (F) Gefitinib CPAP 6 hour (GC6) animals have ERK1/2 positive cells in airway epithelium. (G) Gefitinib Ventilated 6 hour (GV6) animals have no phosphorylated ERK1/2 in bronchioles. (H) Loss of pERK1/2 is still present at 24 hours in GV24 animals. Mean ± SEM. * p < 0.05 vs SC6. Images 20X, Scale bar = 25 nm.

Mechanical ventilation increased Ki67 protein, a marker of cellular proliferation, in the bronchiolar epithelium and the peripheral lung tissue (Fig 2A–2D) at 24 hours in animals receiving saline or Gefitinib. Phosphorylated Erk1/2 (Fig 2E–2H), a mediator of cell growth and differentiation, was present in the bronchiolar epithelial cells of animals receiving CPAP only, but was lost at 6 hours and 24 hours for animals receiving mechanical ventilation. These results also did not differ with Gefitinib. TUNEL assay, which indicates the degree of apoptosis, did not indicate any appreciable degree of apoptosis in any of the groups (data not shown). Animals that received mechanical ventilation (Fig 2C, 2D, 2G and 2H, Table 1) had decreased airway expansion at 30 cmH_2_O fixation than animals that received CPAP (Fig 2B and 2F). In the left mainstem bronchi, mRNA for basal cell markers p63 and KRT8 were increased in mechanically ventilated animals at 6 hours receiving saline, but no difference was found with Gefitinib (Table 2). KRT14 and KRT5 mRNA did not change in any group compared to 6 hour saline CPAP controls. MUC5B mRNA, a precursor to goblet cells, was increased by 24 hours in animals receiving gefitinib and CPAP. MUC5B mRNA increased with mechanical ventilation at 6 hours, with no additional effect of Gefitinib. These changes were small and of questionable relevance.

**Table 2:** Changes in mRNA in left mainstem bronchi and in peripheral lung.

Group	Left Mainstem Bronchi					Peripheral Lung Tissue		
	p63	KRT14	KRT5	KRT8	MUC5b	ErbB2	ErbB3	ErbB4
**Saline CPAP 6h**	1.0+0.2	1.0+0.2	1.0+0.1	1.0+0.1	1.0+0.1	1.0 ± 0.2	1.0 ±0.2	1.0 ± 0.2
**Gefitinib CPAP 6h**	1.3+0.2	1.0+0.2	1.1+0.2	1.1+0.1	1.3+0.1	0.7 ± 0.1	0.7 ± 0.1	0.7 ±0.2
**Saline CPAP 24h**	1.0+0.2	1.1+0.2	0.8+0.1	1.0+0.1	1.4+0.2	0.6 ± 0.1	0.6 ± 0.1	0.5 ± 0.2
**Gefitinib CPAP 24h**	1.5+0.4	1.3+0.2	1.1+0.2	1.6+0.4	1.6+0.1*	0.4 ± 0.1*	0.6 ± 0.2	0.7 ± 0.2
**Saline Vent 6h**	1.9+0.2*	1.4+0.2	1.3+0.2	1.9+0.2*	1.9+0.3*	0.4± 0.1*	0.5 ± 0.1*	0.3 ± 0.2*
**Gefitinib Vent 6h**	1.6+0.2	1.5+0.2	1.1+0.2	1.4+0.2	1.9+0.4	0.4± 0.1*	0.5 ± 0.1*	0.6 ± 0.2
**Saline Vent 24h**	0.8+0.1	0.9+0.1	0.8+0.1	1.0+0.2	1.1+0.2	0.7 ± 0.1	1.0 ± 0.2	1.0 ± 0.1
**Gefitinib Vent 24h**	0.8+0.1	1.2+0.2	1.1+0.3	1.0+0.2	2.0+0.5	0.7 ± 0.1	0.6 ± 0.1	0.8 ± 0.2

* p < 0.05 vs Saline CPAP 6h

#### ErbB family of receptors

mRNA for all four ErbB receptors (EGFR, ErbB2, ErbB3, ErbB4) were detected in the preterm peripheral lung. Based of RT-PCR ΔCq data, normalized to 18S, the relative abundance of each receptor mRNA were determined. Control lambs had 26 fold more ErbB2 mRNA (ΔCq 13.1±0.1) than EGFR mRNA (ErbB1 ΔCq 17.9±0.4). ErbB3 mRNA was 7 times more abundant (ΔCq 14.9±0.2) than EGFR, whereas ErbB4 had 14% the mRNA level (ΔCq 20.7±0.4) of EGFR. Gefitinib decreased ErbB2 at 24 hours compared to saline controls, but the two 24-hour CPAP groups themselves were not significantly different (Table 2). Mechanical ventilation decreased ErbB2 mRNA at 6 hours, but Gefitinib had no effect. ErbB3 mRNA expression decreased at 6 hours post mechanical ventilation in both the saline and Gefitinib groups (Table 2). ErbB4 expression was decreased at 6 hours in ventilated lambs receiving saline compared to saline CPAP animals, but no difference was found between groups receiving Gefitinib and saline.

### Lung inflammation and acute phase activation

**Fig 3:**
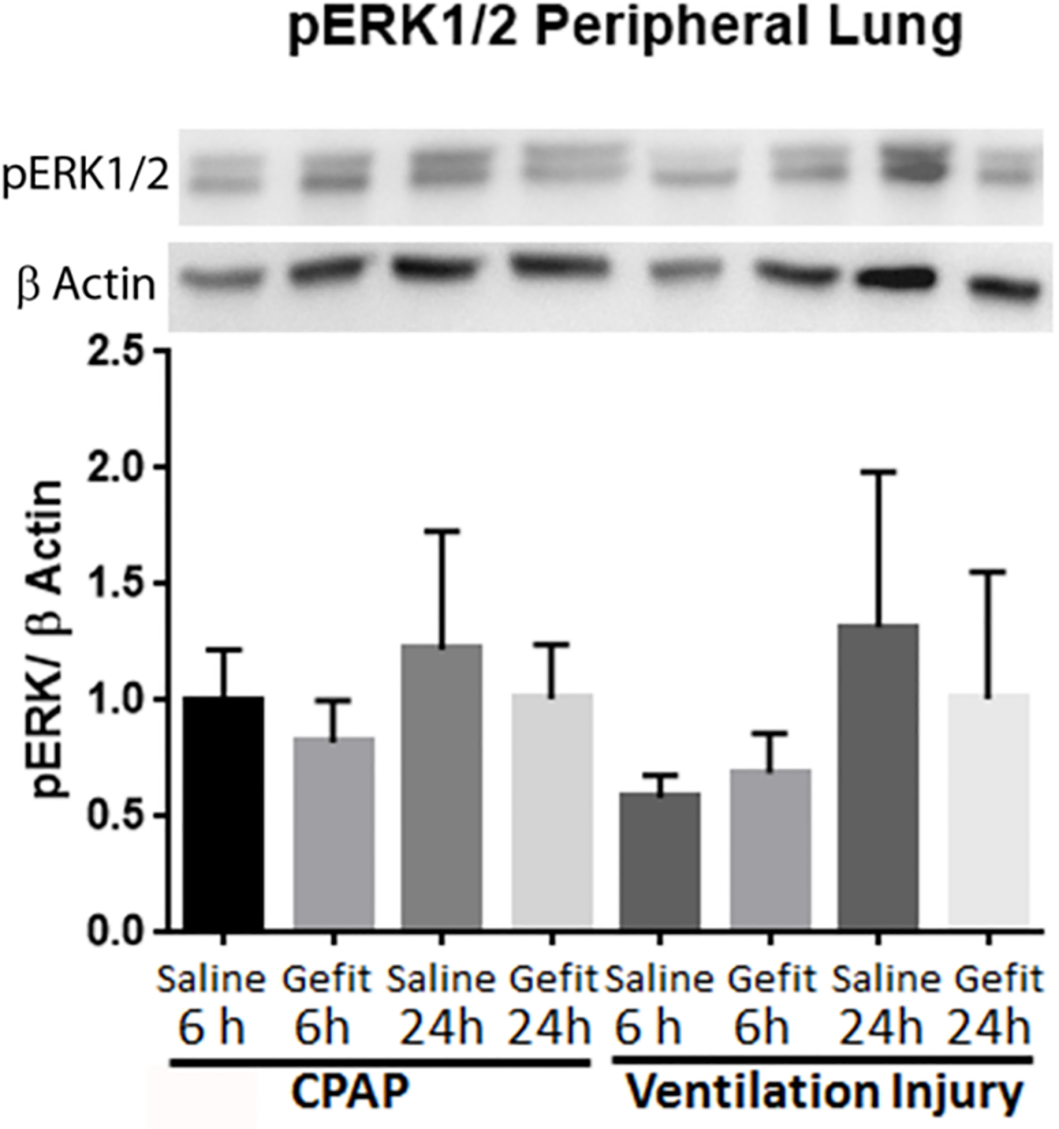
pERK 1/2 protein in lung tissue after mechanical ventilation and Gefitinib exposure. pERK expression divided byβ actin is similar among all groups. Representative blots for pERK 1/2 andβ Actin. Displayed as fold increase over 6 hour Saline CPAP animals. Representative blots for pERK 1/2 andβ Actin. Mean ± SEM, n = 5–6 animals analyzed per group.

Both the saline and Gefitinib ventilated animals had inflammatory cells in the BALF and edema and congestion on histologic exam (Table 3, Fig 2, Table 1 Injury scores). The majority of the inflammatory cells were monocytes in the ventilated lambs at 6 hours (82%) and 24 hours (80%). There was no difference in the percentage of monocytes or neutrophils between the saline and Gefitinib groups. Pro-inflammatory cytokine mRNA for IL-1β, IL-8, and MCP-1 increased with mechanical ventilation in the peripheral lung at 6 and 24 hours (Table 3) in all groups. No difference in these values were noted between groups that received Gefitinib. pERK 1/2 in lung homogenates did not change with mechanical ventilation, CPAP or Gefitinib exposure at 6 hours or 24 hours (Fig 3). pERK after 6 hours in ventilation saline group was non-significantly decreased to 60% of saline CPAP animals (p = 0.08).

**Table 3:** BAL inflammatory cells and lung cytokine mRNA.

Group	BAL Fluid Cells		Peripheral Lung Tissue		
	Neutrophils	Monocytes	IL-1β	IL-8	MCP-1
	Cells/kg x10^5^		mRNA fold change		
**Saline CPAP 6h**	0.3±0.3	0.6±0.4	1.0 ± 0.2	1.0 ± 0.3	1.0 ± 0.5
**Gefitinib CPAP 6h**	0.9±0.6	2.1±1.2	1.6 ± 0.4	1.1 ± 0.2	1.5 ± 0.9
**Saline CPAP 24h**	0.4±0.2	2.5±1.8	4.4 ± 2.0	4.1 ± 2.6	15.3 ± 11.8
**Gefitinib CPAP 24h**	0.0±0.0	0.1±0.1	0.9 ± 0.1	0.8 ± 0.2	2.9 ± 1.0
**Saline Vent 6h**	17±7*	106±54*	4.5 ± 1.2*	6.7 ± 2.1*	11.2 ± 3.4*
**Gefitinib Vent 6h**	24±7*	86±13*	8.2 ± 2.0*	8.7 ± 2.6*	13.5 ± 4.2*
**Saline Vent 24h**	28±13*	148±47*	3.1 ± 0.3*	3.7 ± 0.9*	3.6 ± 0.9
**Gefitinib Vent 24h**	40±10*	140±30*	3.5 ± 0.6*	3.9 ± 0.7*	3.2 ± 0.7

* p < 0.05 vs Saline CPAP 6h

## Discussion

Inhibition of EGFR with Gefitinib, given both intravenously and intra-tracheally, did not alter the inflammatory or acute-phase response to mechanical ventilation in preterm, fetal sheep. The lungs of ventilated animals had similar histologic appearances with lung congestion, epithelial sloughing, and inflammation. By 24 hours, ventilated animals receiving Gefitinib had similar airway activation and proliferation to fetal lambs receiving saline. Animals receiving CPAP did not have lung inflammation or airway activation. The 24 hour period of exposure to Gefitinib did not cause apparent changes in the lung structure of the lambs receiving CPAP alone. Although inhibiting EGFR in rodent models of LPS and mechanical ventilation can decrease lung inflammation, we did not find these positive effects in preterm sheep [17–19, 28].

The alterations with Gefitinib of the EGFR mRNA in the lungs and liver in the CPAP animals indicates that the combination of intravenous and intra-tracheal dosing likely decreased the EGFR signaling in the animals. The unavailability of an antibody for phosphorylated EGFR in sheep is a limitation to confirming this observation. The dosing regimens were based on cell culture and cancer treatment regimens. Since the trachea was ligated in the animals, the 1 mg Gefitinib instilled via the trachea would be distributed into the expected 90 ml (30 mL/kg) of fetal lung fluid to yield a concentration of 11 μg/ml (25μM). This concentration is similar to the 10 to 20 μM concentration that inhibited EGFR activation in cell culture [38, 39]. As the fetal lung fluid was maintained by ligation of the trachea, Gefitinib was in contact with the apical epithelial cells, which are likely the activation site for agonists released into the airways. The IV dose of 15 mg (5 mg/kg) of Gefitinib is higher than the 250 mg dose (about 3.5 mg/kg) given daily for treatment of lung cancer [40]. Although the blood flow is decreased in the fetal lung to 10 to 25% cardiac output, the systemic Gefitinib should have reached the lung parenchyma [41]. The large animal model limits the number of doses and time points that can be evaluated due to expense, thus differences could exist at higher doses. EGFR plays an important role in alveolarization and lung fibrosis, but the length of time after exposure (6 and 24 hours) is too short to evaluate these effects in this gestation for sheep [42]. The lack of an effect on inflammation or airway proliferation in this experiment suggests that blocking EGFR would not be beneficial in preterm infants. The importance of EGFR for lung development makes increasing the dose of Gefitinib impractical [25, 39].

The lack of a response to EGFR inhibition on lung inflammation in fetal sheep may be due to species or gestational age differences between animal models. In rodent models EGFR inhibition caused decreased lung inflammation from mechanical ventilation and LPS exposure, and airway mucous cell proliferation in asthma models [17–19, 28]. However, the distribution of cell types within the airways and peripheral lungs of sheep are closer to humans than rodents [9–11]. EGFR is prominent in basal cells in humans, and activation of EGFR with EGF in cultured bronchiolar cells increased basal cell markers of differentiation (Keratin (KRT) 14+, KRT6+, involucrin) [43]. The increase in p63 and KRT8+ mRNA in these lambs indicate basal cell activation and was consistent with our previous report of increased p63 protein caused by mechanical ventilation of preterm sheep [15]. EGF is low in healthy adults in the airway epithelium, but increases with smoking [43]. We previously were unable to isolate epidermal growth factor (EGF) mRNA from preterm sheep and EGF mRNA is of low abundance in mRNA sequencing of sheep lung (unpublished data). Fetal sheep and monkeys respond to intra-amniotic EGF with airway proliferation, and EGF causes lung maturation in monkeys [44, 45]. The mRNA for AREG and EREG, which are upstream of EGFR inhibition, are consistently increased with 30 minutes of mechanical ventilation in preterm sheep [15, 29]. Amphiregulin has less affinity for EGFR than EGF, and phosphorylation of EGFR occurs at a later time point (7 to 14 days) in cells exposed to AREG versus EGF (hours) [38]. The delay in timing between EGF and AREG could explain the differences between responses in preterm sheep and rodents.

ERK1/2 is phosphorylated in response to activation of EGFR and other receptors. Although we expected phospho-ERK1/2 to increase with mechanical ventilation, the opposite was found in the airway epithelial cells. The lack of change in pERK 1/2 on western blot may signify that although EGFR ligand mRNA are increased with mechanical ventilation that EGFR activation may not occur in the preterm lungs. The epithelial cells of the bronchioles of CPAP animals demonstrated robust pERK 1/2 staining, which was lost in the ventilated animals at 6 hours and 24 hours. Movement of the saline/DMSO liquid along the airways in CPAP animals may have triggered the phosphorylation of ERK 1/2. Activation of ERK 1/2 is necessary for airway development, branching, and maturation [46, 47]. Although cyclical stretch can induce ERK 1/2 phosphoralyzation in alveolar epithelial cells, we did not find this in the preterm lambs [47, 48]. The distribution of the cells without the phsopho-ERK1/2 corresponds to the proliferating cells (Ki67+) of the sheep bronchioles. We previously demonstrated these cells are pro-surfactant B and TTF-1 positive, markers found in club cells [15]. It is unclear what loss of phosphorylated Erk1/2 in the airway epithelium would mean in a developing lung.

The other Erb receptor family members (ErbB2, ErbB3, ErbB4) are present in the preterm sheep lung. The relative expressions in the lung are similar to humans, with ErbB2 and ErbB3 mRNA more abundant than EGFR (ErbB1), whereas ErbB4 mRNA is consistently less [43]. ErbB2 and ErbB3 were found in human fetal lung tissue [49]. The lack of effect of EGFR inhibition was not caused by an increase in the other receptors within the family. The decreases in ErbB2, 3, and 4 mRNA with mechanical ventilation at 6 hours are about two-fold, which may not be biologically important. The ligands for the receptors overlap slightly, with AREG primarily triggering EGFR (ErbB1), whereas EREG or Heparin-binding EGF triggers EGFR, ErbB2, and ErbB4 [50]. Activation of ErbB2 and ErbB3 by neuroregulin-1 causes proliferation of fetal lung epithelial cells, but decreases in surfactant protein A [49]. ErbB4 participates in the late lung development and surfactant production [51]. Similar to targeting EGFR, attempts to block the other ErbB receptors could have deleterious effects in the developing lung.

Inhibition of EGFR activation with Gefitinib did not alter the effects of mechanical ventilation on preterm lambs. Changes in lung and liver EGFR mRNA indicate that we blocked the receptor. The role of EGFR in the lung development makes altering this pathway risky in preterm infants. The overall lack of effect of EGFR inhibition on lung inflammation and airway proliferation would suggest that other pathways should be explored for reducing the chronic airway and lung changes associated with BPD.
